# MAPK-Activated Protein Kinases (MKs): Novel Insights and Challenges

**DOI:** 10.3389/fcell.2015.00088

**Published:** 2016-01-08

**Authors:** Matthias Gaestel

**Affiliations:** Department of Biochemistry, Hannover Medical UniversityHannover, Germany

**Keywords:** p38 MAPK, ERK3/4, common docking motif, macrophage-specific activation, dendritic cells, mouse gene-targeting strategy, Ras-induced senescence, DMBA-induced skin tumors

## Abstract

Downstream of MAPKs, such as classical/atypical ERKs and p38 MAPKs, but not of JNKs, signaling is often mediated by protein kinases which are phosphorylated and activated by MAPKs and, therefore, designated MAPK-activated protein kinases (MAPKAPKs). Recently, novel insights into the specificity of the assembly of MAPK/MAPKAPK hetero-dimeric protein kinase signaling complexes have been gained. In addition, new functional aspects of MKs have been described and established functions have been challenged. This short review will summarize recent developments including the linear motif (LM) in MKs, the ERK-independent activation of RSK, the RSK-independent effects of some RSK-inhibitors and the challenged role of MK5/PRAK in tumor suppression.

## Introduction

Besides phosphorylation of other substrates, ERKs and p38 MAPKs are able to signal further downstream by the activation of so called MAPK-activated protein kinases (MAPKAPKs) (reviewed in Cargnello and Roux, 2011). These downstream kinases are the p90 ribosomal-S6-kinases (RSK1-3), the mitogen- and stress-activated protein kinases MSK1/2, the MAPK-interacting kinases MNK1/2 and the MAPKAP kinases MK2, MK3 and MK5/PRAK (Gaestel, 2006). Specific signaling complexes between MAPK and their target MAPKAPKs exist and are the structural basis for the functional downstream-extension of MAPK cascades. Canonical activation pathways have been defined for the exclusive activation of RSKs by ERK1/2, the exclusive activation of MK2/3 by p38α/β as well as the more promiscuous activation of MNKs and MSKs by both ERKs and p38 and of MK5/PRAK by p38β/ERK3/4. Here, I will discuss novel findings regarding the molecular basis of specific and productive signaling complexes between MAPKs and MAPKAPKs, the non-canonical activation of RSKs and recent challenges arising from off target effects of the widely used RSK inhibitors SL0101 and BI-D1870. Furthermore, the challenge of the anticipated tumor-suppressive function of MK5/PRAK is discussed.

## Novel insights

### The molecular basis for MAPKAPK's specific interaction with MAPKs: Classical D motifs and reverse D-motifs constitute the linear motif (LM)

Specific interactions of MAPKs with their activators and substrates are established via the common docking (CD) motif of MAPKs (D-X_2_-D/E) and the docking (D) motif (R/K-R/K-X_2−6_-Ø-X-Ø) or kinase-interacting motif (KIM) (L/V-X_2_-R/K-R/K-X_5_-L) of the substrate or activator (Tanoue et al., 2000; reviewed in Gaestel, 2008). However, while these interactions fully govern the recognition and phosphorylation of unstructured regions in substrates such as transcription factors, the CD-D-interaction is not completely sufficient for establishing the specificity of binding of MAPKs to important activators and other substrates. The isolated D-motifs of MKK3/6 (p38 specific MAPKK) or MKK1/2 (ERK specific MAPKK) are, for instance, not able to discriminate between p38α and ERK2, but bind to both kinases with comparable affinity (Garai et al., 2012). Further structural analyses have revealed that the CD motif of MAPKs can be divided into the negatively charged CD groove and various further hydrophobic pockets or grooves which are able to interact with the basic core of the D-motif and a pattern of further hydrophobic residues located N terminal to the D-motif designated reverse D (revD) motif (Ø-X-Ø-X_2_-Ø-X_4−6_-Ø-X_2_-R/K-R/K) (Garai et al., 2012). Interestingly, this revD motif allows clear discrimination in binding affinity between p38α and ERK2. While the revD motif of RSK1 displays high affinity to ERK2, its binding affinity to p38α is 20-fold lower. Vice versa, the revD motif of MK2 shows strong affinity to p38α but only weak interaction with ERK2 (Garai et al., 2012).

Only the revD of MNK1, which is activated by both ERK2 and p38α, displays similar affinity to both kinases. Hence, a linear motif (LM) formed by the overlapping D and revD motifs is necessary and sufficient to guarantee specific interaction in the binary MAPK/MAPKAPK complexes such as ERK2/RSK1 and p38α/MK2. The following alignment shows the D-, KIM-, and revD motifs identified in MAPK substrates and activators. Together, these overlapping motifs should be regarded as the linear motif (LM). Ø stands for a hydrophobic amino acid, X_*n*_ for the number n of variable amino acids:

D:                                           R/K-R/K-X_2−6_-Ø-X-Ø
KIM:                         L/V-X_2_-R/K-R/K-X_5_-L
revD: Ø-X-Ø-X_2_-Ø-X_4−6_-Ø-X_2_-R/K-R/K
**LM:**   **Ø-X-Ø-X_2_-Ø-X_4-6_-Ø-X_2_-R/K-R/K-X_2-6_-Ø-X-Ø**

Although the CD motif-LM-interaction is essential for various MAPK/MAPKAPK complexes, the CD-motif of the atypical MAPKs ERK 3 and ERK4 is not sufficient for the activation of MK5/PRAK. Instead, a novel FRIEDE interaction motif in loop L16 C-terminal to the CD-motif is necessary for MK5/PRAK binding of ERK3/4 (Aberg et al., 2009). Interestingly, the L16 FRIEDE motif in ERK3/4 is activated by phosphorylation of the atypical activation loop SEG in an allosteric manner. The FRIEDE motif interacts with the C-terminus of MK5/PRAK and a mutant lacking 50 C-terminal amino acids but still containing the D-domain of MK5/PRAK is unable to bind to ERK3/4 (Aberg et al., 2006). Hence, this interaction is clearly different from the CD-LM-module.

### Primary MAPK/MAPKAPK complexes formed by LM-CD motif interaction

**Figure 1:**
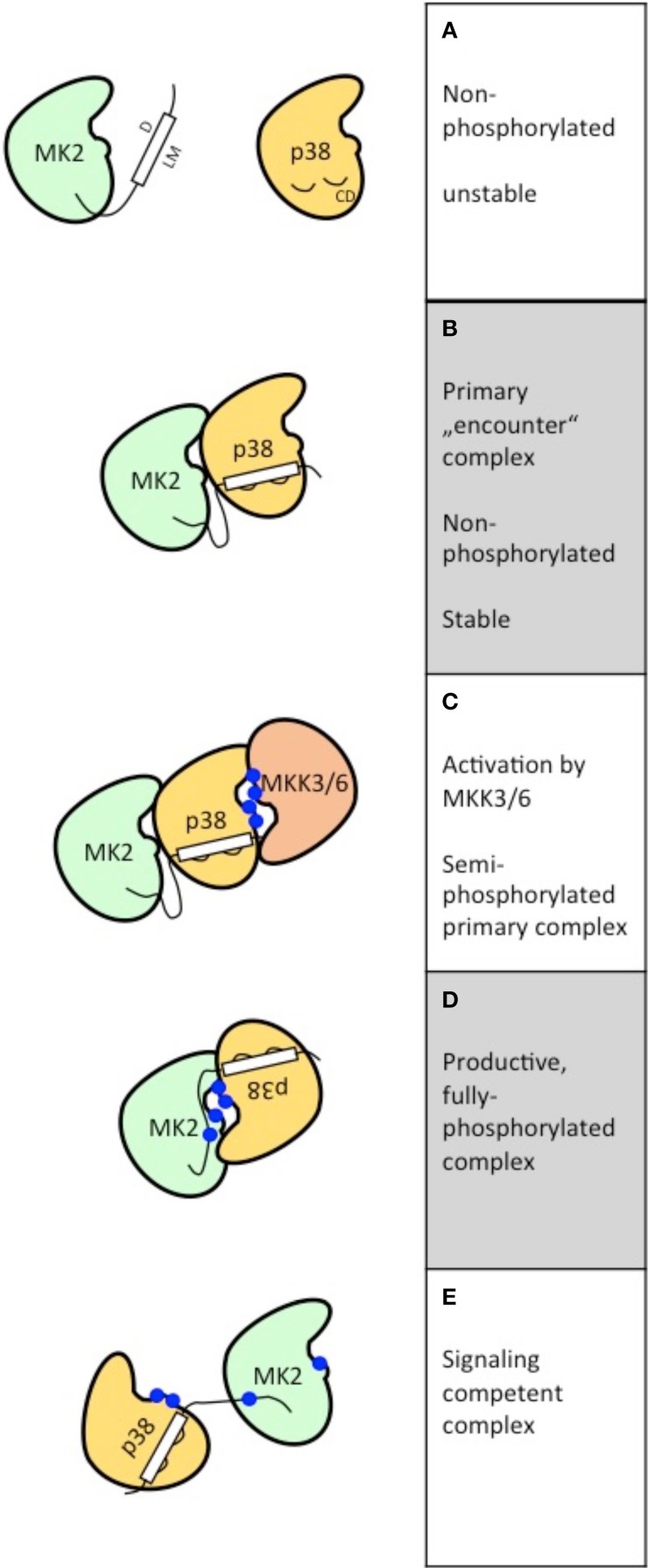
**Schematic representation of the postulated steps to reach signaling competent, fully active binary kinase complexes between MAPKs (here p38α) and MAPKAPKs (here MK2)**. The features of the five different states postulated **(A–E)** are depicted at the right.

The binding of the LM of a MAPKAPK and the CD grooves of MAPKs (Figures 1A,B) is the first step of formation of specific signaling complexes but does not necessarily lead to the formation of a signaling competent complex with both MAPK and MAPKAPK activity. However, the primary “encounter” complex formation (Figure 1B) is already able to cause mutual stabilization of the MAPKs/MAPKAPKs in the specific complex. *In vivo*, this stabilization is reflected by the findings that in non-stimulated MK2-deficient cells the p38α level is significantly reduced (Kotlyarov et al., 2002) and that p38α-deficient resting cells display reduced MK2 levels (Sudo et al., 2005). Furthermore, the formation of primary, non-productive kinase complexes is able to prevent binding to (and activation by) non-specific MAPKs and crosstalk with other signaling pathways. This is demonstrated *in vitro* by the fact that addition of inactive p38α strongly increases the specificity of ppERK2 toward RSK1 and blocks ppERK2's activity against MK2 (Alexa et al., 2015). This finding implies that the stoichiometry between specific MAPKs and MAPKAPKs is an important determinant to maintain the specificity of signaling also *in vivo*. Taking into account that other MAPK substrates and activators compete with MAPKAPKs in binding to the CD motif, signaling complex formation *in vivo* is likely highly sensitive to the local concentrations of these competing interactors. In this regard a high complexity of regulation will also arise due to the fact that local sub-cellular concentrations of many signaling molecules are also signal-regulated. Equal importantly, this result also indicates that artificial overexpression of a specific MAPK or MAPKAPK, which can lead to significant stoichiometric alterations between specific MAPKs and/or MAPKAPKs in the cell, could also lead to artificial activation of non-specific signaling pathways. This would explain the initial observation that MK3, a kinase downstream to p38α/β, is activated by ERKs, JNKs, and p38 in cells overexpressing these MAPKs (Ludwig et al., 1996) or that MK5/PRAK, a kinase activated by the atypical ERK3/4 (see below), also displays docking to p38α when both kinases are overexpressed (New et al., 2003).

The three-dimensional structure of a primary MAPK-MAPKAPK-complex between non-phosphorylated p38α/MK2 has been established (White et al., 2007). In this complex the LM of MK2 is bound to the CD motif of p38α. Both kinases bind in a parallel “head to head” orientation (Figure 1B), but catalytic and substrate regions are distantly located at different sides of the kinase heterodimer making it unlikely that this is a signaling competent complex. However, this orientation would enable upstream activators, such as MKK3 or MKK6, to phosphorylate the activation loop of p38α leading to a semi-phosphorylated primary complex (Figure 1C).

### Productive dimerization leading to active signaling complexes

The three-dimensional structure of another non-phosphorylated MAPK/MAPKAPK complex consisting of ERK2 and RSK1 has recently been determined revealing a structure for a pre-catalytic state of anti-parallel “head to tail” orientation where both kinases face each other and the activation loop of RSK2 is located close to the catalytic center of ERK2 (Alexa et al., 2015). After phosphorylation of ERK2 by the upstream activator MEK1/2 only minor readjustments of the orientation of the binary complex seem necessary to activate RSK1 by phosphorylation of the CTD leading to a productive signaling module (Alexa et al., 2015).

In the case of p38α/MK2 more complex changes in orientation of the molecules in the complex seem necessary to enable p38 to phosphorylate the regulatory sites of MK2 (Figure 1D). It could be assumed that these changes are allosterically induced by phosphorylation of p38α at the activation loop. After phosphorylation of the regulatory sites of MK2 at the activation loop and in the hinge region between catalytic core and C-terminal extension, MK2 itself undergoes a structural transition involving a major conformational change of the atypically structured APE motif of MK2 (Alexa et al., 2015). As a result of this process a fully active signaling complex is formed (Figure 1E). The transition from the primary “encounter” complex to the fully active p38α/MK2 signaling complex is accompanied by a reduction of the affinity of interaction reflected by a increase of the K_*d*_-value from 2.5 nM for non-phosphorylated MK2 and p38α to about 60 nM for phosphorylated MK2 and p38α (Lukas et al., 2004). Interestingly, a number of proteins and cellular structures, such as LIMK1 (Kobayashi et al., 2006), keratin K8/K20, or K8/K18 complexes (Menon et al., 2010) and the neighboring immediate early promoter binding factors CREB/SRF (Heidenreich et al., 1999; Ronkina et al., 2011) are substrates for both p38α and MK2 indicating that the fully active p38α/MK2 complex might act cooperatively to phosphorylate these proteins and structures.

### Non-canonical activation of RSK in dendritic cells

Although there is a specific interaction between ERKs and RSKs via the CD-LM-interaction in many cell types, an ERK-independent but p38α-dependent activation of RSK by MK2 and MK3 has been described in dendritic cells. In these cells MK2/3 bypass phosphorylation of the C-terminal kinase domain (KD) by ERKs by directly phosphorylating the auto-phosphorylation site S386 between N- and C-terminal KD, a prerequisite for the activation of the N-terminal KD by PDK1 (Zaru et al., 2007). Recently, the structural and functional basis for the cell type-specific operation of this alternative activation mechanism of RSKs has been characterized further (Zaru et al., 2014). It has turned out that the non-canonical activation of RSKs is specific for hematopoietic cells, such as dendritic cells and macrophages, and that the C-terminal KD of RSK is dispensable for this activation. Furthermore, the existence of the non-canonical activation mechanism is accompanied by an increased constitutive cytoplasmic localization of p38α/MK2/3 in these cells and a very low activation of ERKs by inflammatory stimuli, such as LPS. Hence, in these cells a certain plasticity of MAPK signaling guarantees the LPS-induced TLR-mediated interferon-β induction via the p38α/MK2/3-RSK-pathway. The interaction between MK2/3 and RSK in these cells seems rather transient (Zaru et al., 2014) and it is not clear whether further cell type-specific protein partners facilitate this interaction in macrophages and dendritic cells.

## Established functions challenged

### Challenged specificity of the compounds BI-D1870 and SL0101 and mTORC1-related function of RSKs

In tests against a panel of recombinant protein kinases the compounds BI-D1870 and SL0101 appeared as relatively specific inhibitors for RSK1 and RSK2 (Bain et al., 2007). However, said panel did not contain mTOR or mTORC1 and a recent study demonstrated that BI-D1870 and SL0101 also modulate mTORC1-p70S6K signaling in different directions (Roffé et al., 2015). Since SL0101 clearly also inhibits mTORC1-p70S6K signaling, the demonstration that RSK phosphorylates ribosomal protein S6, a substrate of p70S6K, using this inhibitor is challenged. Interestingly, BI-D1870 increased p70S6K activation in an ERK1/2- and RSK-independent manner by a mechanism unknown to date. In the light of these findings, the interpretation of the results presented in nearly 100 publications describing effects of these inhibitors without confirming these effects by further experiments, such as knockdown or overexpression of active kinase, should be reassessed. Meanwhile, novel and more specific RSK-inhibitors have also been identified (Jain et al., 2015) enabling us to better define the *in vivo* function of these kinases.

### Challenged function of MK5/PRAK as tumor suppressor

Controversial discussions regarding the activation mechanism and function of MK5/PRAK have been published. As seen from the LM alignment below, the sequence of the LM present in this protein kinase bears similarity to both the LM of RSK and MK2, indicating possible interaction with ERKs or p38 MAPKs:

RSK1:            721-PQLKPIESSILAQRRVRKLPS-741
                   :::.      .. .:   .
MK5/PRAK:  348-VSLKPLHSVNNPILRKRKLLGTK-364
                 . .: .  . ::.: ::. .
MK2:              372-IKIKKIEDASNPLLLKRRKKARA-392
LM:                        **ØXØXXØXXXXXXØXXRRXXXØXØ**
                                **KK**

In line with this similarity, activation of MK5/PRAK has been observed by p38 MAPKs and by ERKs when these kinases were overexpressed in mammalian cells (New et al., 1998; Ni et al., 1998). Furthermore, there is the FRIEDE-binding region (see above) in the C-terminal stretch of 50 amino acids, which enables interaction of this kinase with ERK3/4 (Aberg et al., 2009). Overexpression of both p38α and ERK3/4 leads to phosphorylation of MK5/PRAK at its regulatory site T182 and its activation as measured by phosphorylation of the peptide PRAKtide. While several publications describe a p38-dependent activation of MK5/PRAK (New et al., 1998), others could not detect activation of MK5/PRAK by stimuli, which activate p38 MAPKs, such as arsenite or high osmolarity (sorbitol) treatment (Shi et al., 2003). ERK3/4 activity and binding of the FRIEDE motif to MK5/PRAK can be stimulated by phosphorylation by p21-activated kinase 1 (PAK1) in the SEG motif in the activation loop (De la Mota-Peynado et al., 2011; Déléris et al., 2011) connecting MK5 to signaling of the small GTP-ase Rac. Furthermore, acetylation of MK5/PRAK at lysine K364 in the putative LM has also been described to increase its activity, although it should interfere with binding of the appropriate MAPK (Zheng et al., 2013), and various substrates of MK5/PRAK, such as p53 (Sun et al., 2007), HSP27 (Kostenko et al., 2009), FoxO3a (Kress et al., 2011), Foxo1 (Chow et al., 2013), and Rheb (Zheng et al., 2011) have been proposed.

The function of MK5/PRAK has been mainly characterized by two different mouse knockout approaches targeting exon 6 and exon 8, respectively (Shi et al., 2003; Sun et al., 2007). Surprisingly, it has recently turned out that both knockout approaches for MK5/PRAK failed to delete the entire protein (Ronkina et al., 2015). Instead, two different truncated MK5/PRAK forms are still present in the knockout mice (Table 1). Since these mutants display different biochemical and cellular properties (Table 1) it is not surprising that the effects of expression of these mutants in cellular systems differ: While MEFs from the Δex6-targeted mice did not show altered p21^WAF^ level in response to H-Ras-G12V expression and did not grow in soft agar, Δex8-targeted MEFs displayed reduced levels of the key marker of tumor suppression p21^WAF^ and growth in soft agar (Sun et al., 2007; Ronkina et al., 2015). It is highly probable that the properties of the different MK5/PRAK deletion mutants also contribute to the phenotype of the targeted mice in the established DMBA one-step skin tumor model. Hence, it is not surprising that Δex6-targeted mice did not display increased skin tumor formation in this model, while Δex8-targeted did (Sun et al., 2007; Ronkina et al., 2015). Since the results obtained using the Δex8-targeted mice formed the basis for the formulation of the tumor suppressive role of MK5/PRAK (Sun et al., 2007) as well as for the identification of MK5/PRAK as a tumor-promoting angiogenic factor (Yoshizuka et al., 2012), these roles are challenged and should be revisited by MK5 targeting approaches deleting the entire protein kinase. Once the phenotype of the real MK5/PRAK knockout mouse has been described, these results should serve to clarify the physiological function of MK5/PRAK.

**Table 1:** **Comparison of the results of the targeting approaches for MK5/PRAK**.

	**“MK5 knockout” (Shi et al., 2003)**	**“PRAK knockout” (Sun et al., 2007)**
MK5/PRAK targeting strategy	Deletion of exon 6 (Δex6)	Deletion of exon 8 (Δex8)
Protein	Truncated, deletion of 30 amino acids (131–160)	Truncated, deletion of 27 amino acids (194–220)
Stability	Instable	Stable (similar to WT)
Localization	Cytoplasmic	Nuclear (similar to WT)
Kinase domain	Subdomains VIa, VIb missing	Stretch between subdomains VIII and IX shortened
Kinase activity	Not-detectable	Residual autophosphorylation
Reduction of H-Ras-G12V-induced p21^WAF^ expression in targeted MEFs	−	+
Ras-induced tumorigenity/growth of targeted MEFs in soft agar	−	+
Increased skin tumor formation in the one step DMBA model in the targeted mouse strain	−	+

## Funding

The work of MG was funded by Deutsche Forschungsgemeinschaft.

### Conflict of interest statement

The author declares that the research was conducted in the absence of any commercial or financial relationships that could be construed as a potential conflict of interest.
